# Editorial: Unveiling host-pathogen interactions: insights into animal cellular immunity and novel diagnostics

**DOI:** 10.3389/fcimb.2024.1543349

**Published:** 2025-01-15

**Authors:** Haojie Wang, Qiang Zhang, Jiangchao Zhao, Changqing Yu, Changyou Xia, He Zhang

**Affiliations:** ^1^ State Key Laboratory for Animal Disease Control and Prevention, Harbin Veterinary Research Institute, Chinese Academy of Agricultural Sciences, Harbin, China; ^2^ College of Veterinary Medicine, Huazhong Agricultural University, Wuhan, China; ^3^ Animal Science, University of Arkansas, Arkansas, AR, United States; ^4^ Yibin Vocational and Technical College, Yibin, China

**Keywords:** pathogen, host, molecular mechanisms, diagnostic technologies, animal diseases, vaccines

We conducted this Research Topic to explore the interaction between pathogens and host cells, revealing how pathogens achieve cellular infection by precisely interacting with host cell proteins. This Research Topic further elucidates how host cells play a crucial role in immune defense, blocking pathogen invasion and hindering its replication and expansion within cells. Pathogen infection is one of the most serious factors threatening the development of the livestock industry (Sollner et al., 2021; Li et al., 2024). In-depth exploration of the molecular mechanisms underlying pathogen-host interactions will provide a solid foundation for pathogen control and offer critical theoretical support for the development of antiviral drugs, vaccines, and therapeutic antibodies (Muzykina et al., 2024; Shan et al., 2024; Zheng et al., 2024). Through more refined research into intracellular co-evolution mechanisms, we will be able to design more effective diagnostic methods and preventive measures. In the field of animal disease diagnosis, numerous new diagnostic technologies have been applied to clinical diagnostics in recent years, but issues such as low sensitivity, poor accuracy, and slow detection speed remain. There is a continuing need to explore new diagnostic technologies, including both pathogen-based and antibody-based diagnostic techniques, which can offer faster, more accurate, and more convenient methods for clinical diagnosis of outbreaks. Currently, vaccine prevention remains the primary means of controlling animal diseases (Shan et al., 2024). Although vaccines for various animal diseases have been developed, many still suffer from poor safety and low efficacy, making the development of novel immune strategies a significant challenge for researchers in the field of animal disease prevention and control (Tang et al., 2024). This special Research Topic, published in Frontiers in Cellular and Infection Microbiology, represents an important step forward in understanding the themes outlined above. Our Research Topic includes 20 key studies and focuses on three major areas: the immune mechanisms of pathogen-host interactions, the development of new diagnostic technologies for animal diseases, and the development of novel vaccines for animal diseases.

## Immune mechanisms of pathogen-host interaction

The interaction between pathogens and host cells plays a central role in infection, immune evasion, and pathogenicity (Yu et al., 2023). Xu et al. demonstrated that *Pasteurella multocida* (*P. multocida*) interacts with the TLR2 receptor on host macrophages, activating the NF-κB and ERK1/2 signaling pathways to promote the secretion of pro-inflammatory cytokines, facilitating pathogen growth and spread. The study confirmed that the recombinant protein PMCNA_RS00975 from *P. multocida* activates NF-κB and ERK1/2 through TLR2, contributing to the virulence of *P. multocida*. Huang et al. confirmed that tuberculosis (TB), caused by *Mycobacterium tuberculosis* (Mtb), is a fatal infectious disease, and its progression involves complex interactions between the host immune system and Mtb. IL-26, a member of the IL-10 family, has antimicrobial and pro-inflammatory functions. They found that IL-26 mRNA levels were significantly elevated in peripheral blood mononuclear cells from active TB patients, while plasma levels of IL-26 were lower. IL-26 stimulation of THP-1 cells promoted the polarization of M1 macrophages, ROS production, and intracellular killing of Mtb. This study clarified the role of IL-26 in tuberculosis and provided a theoretical basis for new therapeutic strategies. These findings offer new insights into the interactions between pathogens and host immune responses and identify potential targets for immune intervention.

## Development of novel diagnostic technologies for animal diseases

This special Research Topic also focuses on the establishment of novel diagnostic methods for pathogen detection. With the increasing diversity and prevalence of pathogens, traditional diagnostic methods face challenges such as low sensitivity and poor specificity. Real-time quantitative PCR technologies, such as TaqMan PCR, have become essential tools for monitoring pathogens in pigs and poultry due to their high sensitivity, specificity, and speed. Wang et al. developed a quadruple TaqMan real-time quantitative PCR method that can simultaneously detect gastrointestinal pathogens in pigs (e.g., *Salmonella*, *Escherichia coli*, *Lawsonia intracellularis*, and *Brachyspira hyodysenteriae*), significantly improving diagnostic efficiency, avoiding cross-reactions, and meeting clinical and epidemiological needs. Wang et al. developed a quadruple TaqMan real-time quantitative PCR method to detect four similar pathogens in pigs, helping to avoid misdiagnosis or missed diagnosis due to similar clinical symptoms. Furthermore, the detection and genotyping of various pathogens, such as *Streptococcus suis* and Duck circovirus, provide rapid and accurate tools to facilitate epidemic control. The Research Topic also reports on recent advances in antibody detection methods for animal diseases. In recent years, antibody detection methods have been continuously innovated, with enzyme-linked immunosorbent assay (ELISA) widely used to monitor the immune status of pig populations and disease outbreaks. The regional outbreak of SADS-CoV in China has caused significant economic losses and poses a threat to the pig industry. Despite molecular epidemiological surveys, there is a lack of long-term data and extensive serological studies. Liu et al. developed an indirect ELISA based on the SADS-CoV S1 protein to evaluate 978 pig serum samples from 29 provinces in China between 2022 and 2023. The results showed an overall seroprevalence of 59.97%, with provincial seroprevalence ranging from 16.7% to 77.12%, and monthly seroprevalence ranging from 42.61% to 68.45%. Higher seroprevalence was observed in Northeast, North China, and Central China regions, with the highest in spring and the lowest in autumn. These findings provide important data for evaluating the epidemiology of SADS-CoV, formulating control measures, and developing vaccines.

## Development of novel vaccines for animal diseases

Finally, the Research Topic highlights the importance of vaccine and antibody research in animal disease control. The development of subunit vaccines and monoclonal antibodies offers new strategies for controlling animal diseases. *Mycoplasma synoviae* (MS) is a significant avian pathogen that causes infectious synovitis and respiratory diseases, leading to substantial economic losses. Vaccination is an effective method for preventing MS infections, but commercial subunit vaccines are still challenging to develop. Sun et al. isolated six MS strains from different provinces in China, performed whole-genome sequencing, and identified 22 common genes as potential antigenic targets. Through antibody epitope prediction, they selected 10 candidate vaccine proteins and eventually developed a multivalent subunit vaccine composed of MSPB, Ppht, Cfba, and EF-G, with an immune protection rate of 90–100%, providing a new approach for MS vaccine development. Liu et al. achieved soluble expression of the recombinant protein P32 from Goatpox virus (GTPV) and developed specific monoclonal antibodies. By optimizing the P32 gene sequence and synthesizing the *P32Δ* gene, they expressed and purified the soluble recombinant protein rP32Δ in a prokaryotic expression system. Using rP32Δ to immunize mice, they established an indirect ELISA method and selected three hybridoma cell lines secreting monoclonal antibodies against rP32Δ, namely 2F3, 3E8, and 4H5. These monoclonal antibodies exhibited good specificity, with 3E8 specifically recognizing GTPV in cells. This research provides a foundational material for establishing GTPV detection methods.

## Conclusion

As the challenges of animal disease control continue to grow, research on pathogen-host immune evasion mechanisms, rapid diagnostic technologies, and vaccine and antibody development will be critical areas of focus. We hope that further exploration of pathogen immune evasion mechanisms will reveal the multi-dimensional roles of host immune responses, providing more precise targets for immune intervention strategies. In pathogen detection, there is a need to improve multiplex detection methods and enhance their application, especially for efficient diagnosis and control during outbreaks. In vaccine and antibody research, attention should be given to personalized and precision treatments, developing safer and more effective vaccines and antibodies to provide stronger guarantees for animal health and food safety. This Research Topic covers the latest advances in pathogen-host interaction research, novel pathogen detection methods, and vaccine development, providing a theoretical foundation and technical support for the development of new vaccines and disease detection. It contributes to the prevention and monitoring of animal infectious disease transmission, reduces economic losses, and promotes the development of the livestock industry.
